# Evaluation of factors associated with interhospital transfers to pediatric and adult tertiary level of care: A study of acute neurological disease cases

**DOI:** 10.1371/journal.pone.0279031

**Published:** 2022-12-14

**Authors:** Stanca Iacob, Yanzhi Wang, Susan C. Peterson, Sven Ivankovic, Salil Bhole, Patrick T. Tracy, Patrick W. Elwood

**Affiliations:** 1 Department of Neurology, University of Illinois College of Medicine at Peoria, Peoria, Illinois, United States of America; 2 Illinois Neurological Institute, OSF HealthCare System, Peoria, Illinois, United States of America; 3 Research Services, Department of Internal Medicine, University of Illinois College of Medicine at Peoria, Peoria, Illinois, United States of America; 4 Healthcare Analytics, OSF HealthCare System, Peoria, Illinois, United States of America; 5 Department of Neurosurgery, University of Illinois College of Medicine at Peoria, Peoria, Illinois, United States of America; Policlinico Riuniti of Foggia: S.C. Neurologia Ospedaliera, ITALY

## Abstract

**Introduction:**

Patient referrals to tertiary level of care neurological services are often potentially avoidable and result in inferior clinical outcomes. To decrease transfer burden, stakeholders should acquire a comprehensive perception of specialty referral process dynamics. We identified associations between patient sociodemographic data, disease category and hospital characteristics and avoidable transfers, and differentiated factors underscoring informed decision making as essential care management aspects.

**Materials and methods:**

We completed a retrospective observational study. The inclusion criteria were pediatric and adult patients with neurological diagnosis referred to our tertiary care hospital. The primary outcome was potentially avoidable transfers, which included patients discharged after 24 hours from admission without requiring neurosurgery, neuro-intervention, or specialized diagnostic methodologies and consult in non-neurologic specialties during their hospital stay. Variables included demographics, disease category, health insurance and referring hospital characteristics.

**Results:**

Patient referrals resulted in 1615 potentially avoidable transfers. A direct correlation between increasing referral trends and unwarranted transfers was observed for dementia, spondylosis and trauma conversely, migraine, neuro-ophthalmic disease and seizure disorders showed an increase in unwarranted transfers with decreasing referral trends. The age group over 90 years (OR, 3.71), seizure disorders (OR, 4.16), migraine (OR, 12.50) and neuro-ophthalmic disease (OR, 25.31) significantly associated with higher probability of avoidable transfers. Disparities between pediatric and adult transfer cases were identified for discrete diagnoses. Hospital teaching status but not hospital size showed significant associations with potentially avoidable transfers.

**Conclusions:**

Neurological dysfunctions with overlapping clinical symptomatology in ageing patients have higher probability of unwarranted transfers. In pediatric patients, disease categories with complex symptomatology requiring sophisticated workup show greater likelihood of unwarranted transfers. Future transfer avoidance recommendations include implementation of measures that assist astute disorder assessment at the referring hospital such as specialized diagnostic modalities and teleconsultation. Additional moderators include after-hours specialty expertise provision and advanced directives education.

## Introduction

Interhospital transfer numbers within the United States have increased significantly in recent years. In large healthcare systems, patients are often transferred to tertiary care centers from smaller community hospitals with low-volume emergency departments. It is generally accepted that state-of-the-art diagnostic methods, subspecialty care, interdisciplinary health care teams, 24 hours resident physician coverage and evidence-based medicine are supportive of superior clinical outcomes. Patient preferences and referring and accepting physicians’ underscored need for a specific test or procedure appear to validate critical care referrals [1]. Additional frequently cited reasons are hospital characteristics and compliance with EMTALA guidelines [2, 3]. Current data on neurological cases address mostly traumatic and non-traumatic disease with high transfer rate: non-traumatic intracranial hemorrhage (39.0%), neoplasm (15.8%), trauma (11.9%) and spine disease (9.8%), among others [4]. As the demand for more specialized neurosurgical care centralizes in tertiary care centers so do patient transfers. Up to 35% emergency department (ED) and 10% inpatient unit transfers had surgery within 24 hours [5]. However, lower level hospitals within regional trauma systems often refer minimally injured patients to the Level I Trauma Center, thus overexposing patients to “secondary over-triage,” *e*.*g*., patient over-triage reached a 26% rate, including 24% adult and 49% pediatric referrals with mostly head, neck, and skin and soft tissue injuries [6]. Pediatric referrals of brain trauma cases without associated systemic injury included up to 70% cases that could be treated at referring units [7]. Of non-displaced skull fractures or negative CT scan cases not requiring surgery, 86% were discharged the following day. Observational data from the US Nationwide Emergency Department Sample identified referral rate increases over time, however data suggested transfers of patients with small subcortical or lacunar strokes might be avoided if evaluated at the “spoke” center under “hub” guidance [8].

Data on additional non-traumatic neurological subspecialties is sparse. In the current milieu of increased life expectancy, the burden of neurological disease in the aged population particularly when chronic and associated with co-morbidities mandates efficient patient triage. However, interhospital transfers, although an important link in the continuum of care, are frequently unwarranted. Undoubtedly, disease acuteness weighs heavily on transfer decisions and risk attributed to age, time constraints and referring hospital infrastructure affect transfer dynamics with unwarranted clinical outcomes. Perceived accepting unit subspecialty expertise and quality of care, miscommunication and misdiagnosis add to encumbering consequences. As well, unwarranted transfers increase the burden of health care related costs. Implicitly, patient referral strategies are complex.

This study aims to identify potentially avoidable referrals to pediatric and adult neurological subspecialties, and characterize predictors of unwarranted transfers. Herein, we also ascertain provider underscored strategies to address interhospital transfer process opportunities within our healthcare system.

## Materials and methods

### Patient selection criteria

We performed a retrospective observational review of patients with neurological diagnosis chronologically admitted over two years. The Peoria Institutional Review Board, Peoria, IL (Peoria IRB) approved the study. The need for consent was waived by the ethics committee. The neurosciences service line of our tertiary care hospital with Level I Trauma Center designation and the Children’s Hospital of Illinois (CHOI) provide specialized care in more than 16 subspecialties. Patient interhospital transfers were referred from within the healthcare system or from out-of-system hospitals.

Pediatric and adult patient cohorts were selected based on the International Classification of Diseases, Ninth Revision (ICD-9) diagnosis of neurological disease. Fourteen primary disease categories were analyzed. The category labelled “other” included patients without definitive neurologic diagnosis. Patient demographic characteristics, referring hospital name and location, accepting hospital admission and discharge time, disease category, diagnoses established at both referring and accepting hospitals and reasons a transfer was considered acceptable were noted. Additional transfer process pertaining data (advanced directives, referring hospital resources, condition manageable at the referring institution) were also assessed. Interhospital transfers were classified into justified and potentially avoidable. The primary outcome was potentially avoidable transfer. Transferred cases classified into potentially avoidable had admitting hospital length of stay of up to 24 hours. Patients did not undergo neurosurgery, neuro-interventional procedures, specialized neurological consult and diagnostic modalities, or interventions and consult in specialties other than neurology during their hospital stay. Variables included patient demographics (age, gender, race), diagnosis, insurance, in-system or out-of-system hospital designation, hospital size and teaching status.

### Statistical analysis

Descriptive statistical analysis evaluated sociodemographic (age, gender, race, insurance) data, disease categories and referring facility characteristics. Mean and standard deviation (SD) were reported for continuous variables and percentage for categorical variables. Descriptive statistics (frequency, median, minimum, maximum) were used to summarize the data. The Student’s *t*-test and nonparametric equivalent test (when appropriate) evaluated continuous variables, the Chi-square test or exact Chi-square test categorical variables. To assess age, gender, race, disease category and sending facility associations with the primary outcome, univariate logistic analysis was first performed for all variables, followed by multiple logistic regression analysis. All covariates with p < 0.25, as determined with univariate analysis, were included in the multiple logistic regression analysis with stepwise selection to construct a final model to assess odds of potentially avoidable transfers. We selected 50–59 years and hemorrhagic stroke as referent values for age and disease category based on the evaluation of proportions of avoidable transfers attributed to all age groups and disease categories, respectively. Pairwise comparisons of discrete age groups and disease categories were accounted with the Bonferroni correction method. In the final model, p < 0.05 is considered statistically significant. Confidence intervals were expressed at 95% [95% CI]. Statistical analysis was performed with Statistical Analysis System software version 9.4 (SAS Institute Inc., Cary, NC).

## Results

We analyzed 3539 patient interhospital transfers referred from within the healthcare system and from out-of-system hospitals, over 2 years. Of these, 44.8% and 46% respectively were considered potentially avoidable transfers. Mean patient age at admission was 55.5 ± 25.6 years, 1820 were male patients. The majority of referred cases were Caucasian patients, which showed a lesser proportion of potentially avoidable (46%) compared to justified transfers. We did not identify statistically significant associations between patient sex (p = 0.210) and race (p = 0.484) with the primary outcome (Table 1).

**Table 1:** Potentially avoidable transfers: Patient sociodemographic and hospital characteristics.

Variable	Total	Avoidable	Justified	p-value
**Age**				
N	3539	1615	1924	<0.001^T^
Mean ± SD	55.5 ± 25.6	53.7 ± 27.6	57.0 ± 23.6	
Median (min-max)	61.0 (0–103)	60.0 (0–103)	62.0 (0–103)	
**Age group**				<0.001^c^
0–9	290	169 (58.3)	121 (41.7)	
10–19	166	92 (55.4)	74 (44.6)	
20–29	216	119 (55.1)	97 (44.9)	
30–39	225	105 (46.7)	120 (53.3)	
40–49	293	125 (42.7)	168 (57.3)	
50–59	503	195 (38.8)	308 (61.2)	
60–69	576	230 (39.9)	346 (60.1)	
70–79	634	256 (40.4)	378 (59.6)	
80–89	512	239 (46.7)	273 (53.3)	
90–103	124	85 (68.5)	39 (31.5)	
**Diagnosis**				<0.001^c^
Ischemic stroke	872	275 (31.5)	597 (68.5)	
Hemorrhagic stroke	310	69 (22.3)	241 (77.7)	
Cranial trauma	573	315 (55.0)	258 (45.0)	
Spinal trauma	242	117 (48.3)	125 (51.7)	
Primary tumor	141	45 (31.9)	96 (68.1)	
Metastatic tumor	16	7 (43.8)	9 (56.3)	
Seizure disorders	386	214 (55.4)	172 (44.6)	
Migraine	127	97 (76.4)	30 (23.6)	
Spondylosis	112	59 (52.7)	53 (47.3)	
Dementia	111	57 (51.4)	54 (48.6)	
Infection	81	29 (35.8)	52 (64.2)	
Psychiatric disease	61	36 (59.0)	25 (41.0)	
Autoimmune disease	34	22 (64.7)	12 (35.3)	
Movement disorders	32	21 (65.6)	11 (34.4)	
Neuro-ophthalmic disease	22	19 (86.4)	3 (13.6)	
Hydrocephalus	19	9 (47.4)	10 (52.6)	
Other	400	224 (56.0)	176 (44.0)	
**Sex**				0.210^c^
Female	1719	803 (46.7)	916 (53.3)	
Male	1820	812 (44.6)	1008 (55.4)	
**Race**				0.484^c^
Asian	18	9 (50.0)	9 (50.0)	
Black or African American	167	66 (39.5)	101 (60.5)	
Other	116	52 (44.8)	64 (55.2)	
Unknown	19	7 (36.8)	12 (63.2)	
White or Caucasian	3219	1481 (46.0)	1738 (54.0)	
**Discharge Year**				<0.001^c^
2014	1793	875 (48.8)	918 (51.2)	
2015	1746	740 (42.4)	1006 (57.6)	
**Insurance**				0.0287^c^
Commercial	165	75 (45.45)	90 (54.55)	
Managed Care	773	343 (44.37)	430 (55.63)	
Medicaid	753	383 (50.86)	370 (49.14)	
Medicare (+ Part C)	1758	773 (43.97)	985 (56.03)	
Self-pay/Charity	88	39 (44.32)	49 (55.68)	
**Sending Facility**				0.503^c^
Out-of-system	2476	1139 (46.0)	1337 (54.0)	
OSF HealthCare System	1063	476 (44.8)	587 (55.2)	
**Hospital size**				0.509^c+^
Small	2766	1283 (46.4)	1483 (53.6)	
Medium	639	291 (45.5)	348 (54.5)	
Large	2	0 (0.0)	2 (100)	
Missing	132	41	91	
**Teaching status**				0.011^c^
No	1991	950 (47.7)	1041 (52.3)	
Yes	1272	549 (43.2)	723 (56.8)	
Missing	276	116	160	

Continuous variables were analyzed with the Student’s *t*-test (^T^) and categorical variables with the Chi-square test (^C^). Avoidable and justified transfers are expressed as Total (%). P < 0.05 is considered statistically significant.

Insurance coverage mix showed a greater transfer proportion of Medicare insured patients however, compared to other insurance types this patient group had the lowest potentially avoidable transfer fraction (43.97%). Medicaid insured patients had a lower transfer proportion but the greatest fraction of potentially avoidable transfers (50.86%). Associations between insurance type and primary outcome were significant (p = 0.028). Discharges from the accepting hospital showed significant differences over two years (p < 0.001). Associations of referring hospital size and affiliation with the primary outcome were not significant (Table 1).

Age distribution revealed a maximum of interhospital transfers for the 70–79 years old patient group. The highest fraction of potentially avoidable transfers was determined for the patient group aged over 90 years.

**Fig 1:**
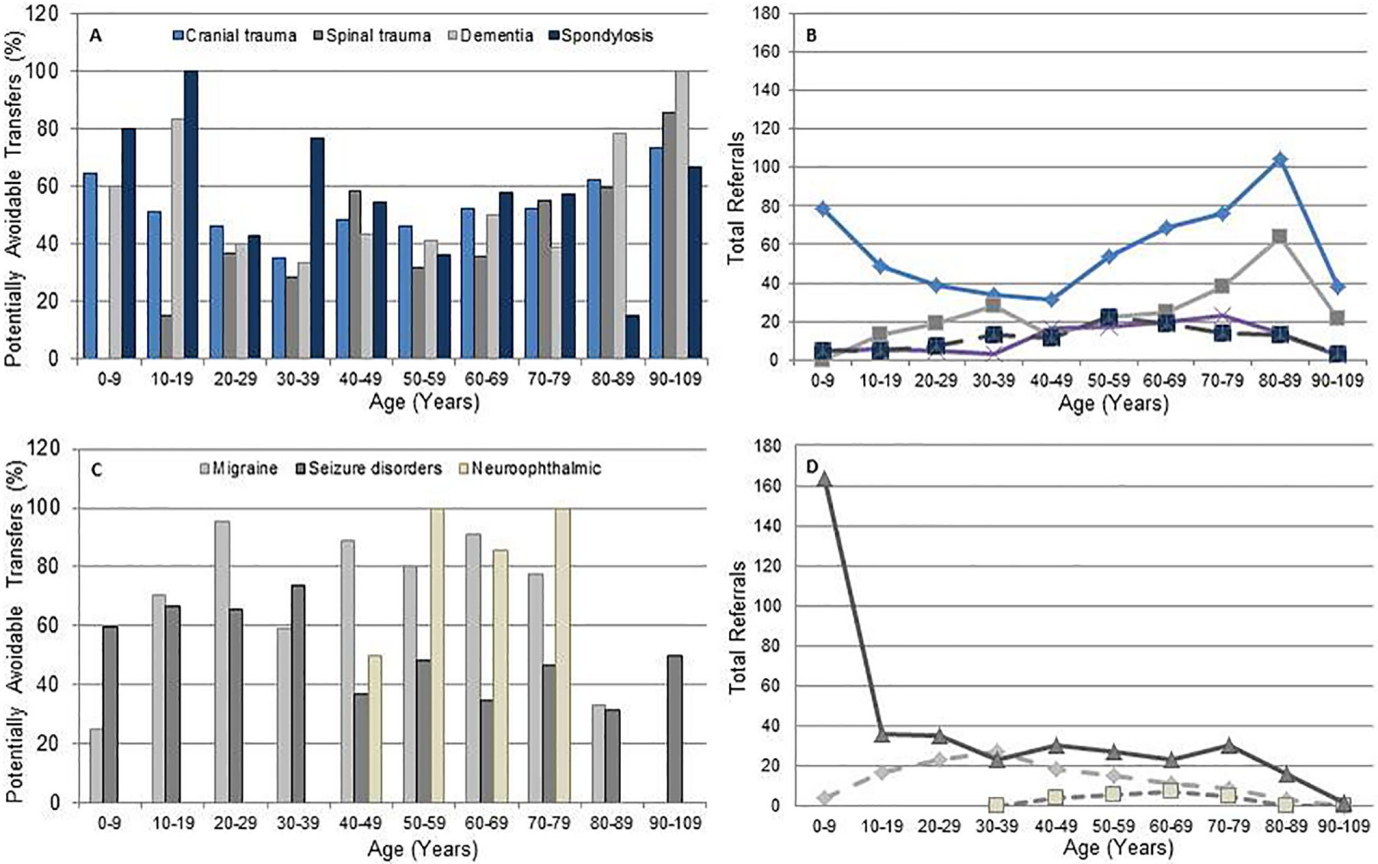
Potentially avoidable transfer proportion correlated with trends in total referrals. (A) Disease categories with high probability of potentially avoidable transfers. (B) Corresponding total referrals showing increasing trends with advancing age. (C) Disease categories associated with significant probability of potentially avoidable transfers. (D) Corresponding total referrals showing declining trends with advancing age.

Age significantly increased the probability of avoidable referrals. Adults over 90 years old diagnosed with trauma or dementia were more likely to be unjustifiably transferred. Interestingly, although a diagnosis of dementia resulted in an elevated potentially avoidable transfer fraction in the pediatric group, a spondylosis case was most likely to be deemed an unjustified transfer. Referrals of trauma, dementia and spondylosis cases showed increasing trends over two years with advancing age, as well as increased proportions of potentially avoidable transfers suggesting that unwarranted referral numbers directly correlate with referral trends. An inverse correlation was determined for migraine, neuro-ophthalmic disease and seizure disorders where total referral trends decreased with advancing age (Fig 1).

The association of these discrete diagnoses with odds of potentially avoidable transfers was significant. When correlated with age, a higher probability of potentially avoidable transfers was determined for patients 0–9 years (OR, 1.48), 80–89 years (OR, 1.55) and over 90 years old (OR, 3.71) (Table 2).

**Table 2:** Probability of potentially avoidable transfers.

Variable	Adjusted OD	95% CI	p-value
**Age**			
0–9	1.48	1.06; 2.06	0.022
10–19	1.30	0.89; 1.89	0.170
20–29	1.27	0.90; 1.78	0.171
30–39	0.95	0.67; 1.32	0.741
40–49	1.00	0.73; 1.36	0.995
50–59	*Referent*		
60–69	1.14	0.88; 1.47	0.323
70–79	1.15	0.89; 1.47	0.293
80–89	1.55	1.19; 2.01	0.001
90–103	3.71	2.40; 5.75	<0.001
**Disease category**			
Autoimmune disease	6.92	3.24; 14.76	<0.001
Dementia	3.83	2.41; 6.08	<0.001
Hydrocephalus	3.08	1.19; 7.95	0.020
Infection	2.04	1.20; 3.47	0.008
Migraine	12.50	7.58; 20.63	<0.001
Movement disorders	6.39	2.91; 14.00	<0.001
Neuro-ophthalmic disease	25.31	7.26; 88.22	<0.001
Other	4.71	3.36; 6.60	<0.001
Psychiatric disease	5.65	3.15; 10.15	<0.001
Seizure disorders	4.16	2.90; 5.97	<0.001
Spondylosis	4.08	2.57; 6.48	<0.001
Hemorrhagic stroke	*Referent*		
Ischemic stroke	1.59	1.17; 2.16	0.002
Cranial trauma	3.96	2.88; 5.46	<0.001
Spinal trauma	3.02	2.08; 4.39	<0.001
Metastatic tumor	2.93	1.05; 8.18	0.040
Primary tumor	1.75	1.12; 2.74	0.014

“Other” denotes disease categories without definitive diagnoses. P < 0.05 is considered statistically significant. 95% CI denotes 95% confidence interval.

When hospital teaching status was factored into the analysis, not only ages over 90 years, but 0–29 years and 80–89 years also correlated with significantly higher odds (Table 3).

**Table 3:** Probability of potentially avoidable transfers correlated with hospital characteristics.

Variable	Adjusted OD	95% CI	p-value
**Age**			<0.001
0–9	1.54	1.07; 2.20	0.019
10–19	1.60	1.05; 2.45	0.029
20–29	1.45	1.01; 2.08	0.042
30–39	0.97	0.69; 1.39	0.885
40–49	1.02	0.74; 1.41	0.895
50–59	*Referent*		
60–69	1.20	0.92; 1.56	0.183
70–79	1.11	0.85; 1.44	0.442
80–89	1.55	1.18; 2.03	0.001
90–103	3.80	2.42; 5.96	<0.001
**Teaching Hospital**			
No vs. Yes	1.27	1.09; 1.47	0.002

Most unwarranted transfers originated from small-sized hospitals (46.4%), while referrals from large-sized hospitals were all justified.

**Fig 2:**
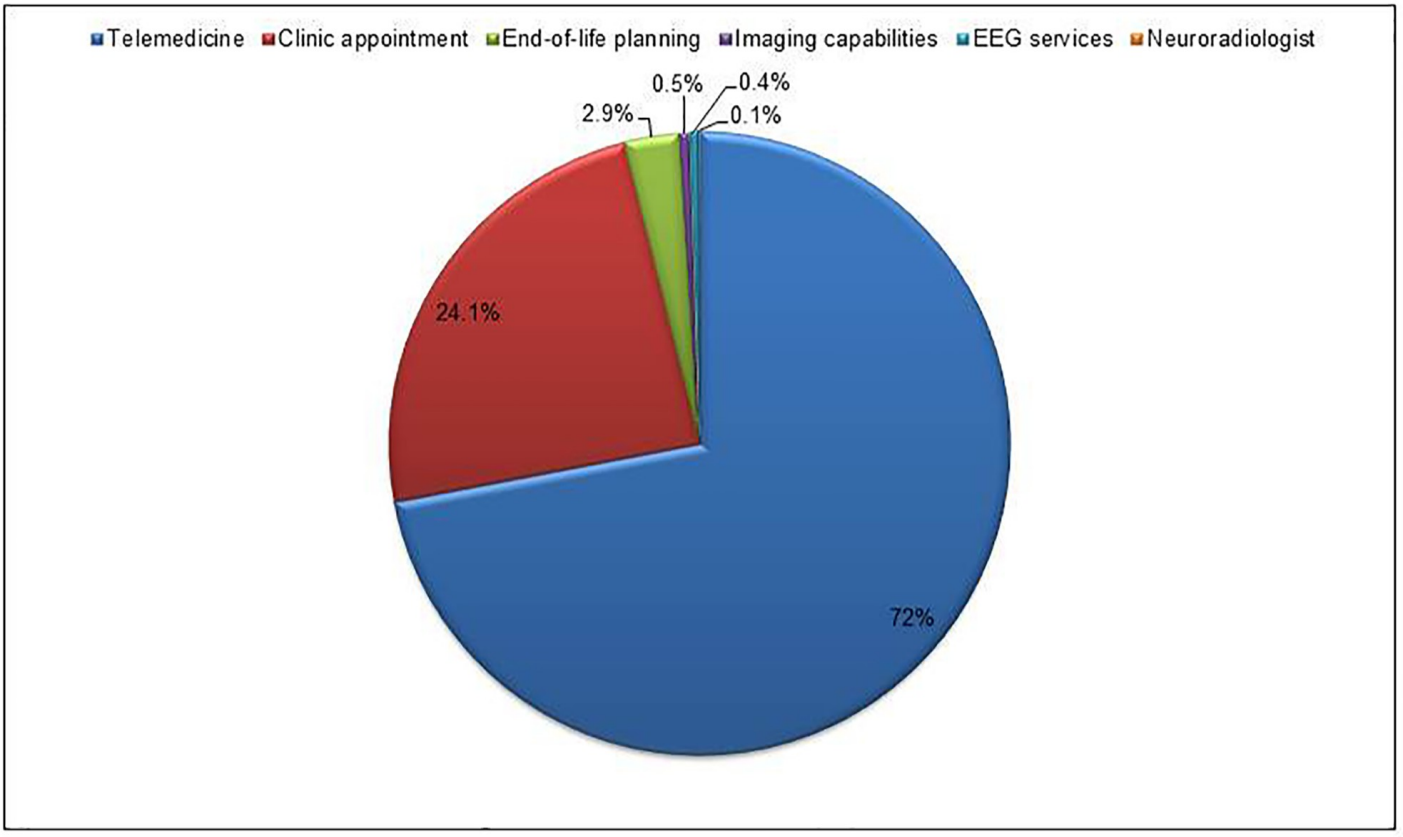
Recommendations and mechanisms in support of future unjustified transfer avoidance.

Support mechanisms that might circumvent an interhospital transfer by virtue of diagnostic workup performed at the referring hospital include teleconsultation at the referring unit (72%), improved infrastructure at low-volume hospitals through implementation of after-hours imaging capabilities (0.5%) and EEG services (0.4%) and specialized consult by the neuro-radiologist. Prompt referral to the neurologic clinic for follow-up (24.1%), palliative course assessment at the referring facility and advanced directive education (2.9%) were also indicated (Fig 2).

## Discussion

Our study evaluates referrals of pediatric and adult patients to 16 neurological subspecialty services in a tertiary care hospital with Level I Trauma Center designation and determines the probability of potentially avoidable transfers. Associations with age and hospital characteristics were evaluated for discrete diagnoses. Interhospital transfers of patients diagnosed with migraine or seizure disorders were deemed potentially avoidable also, cases of neuro-ophthalmic disease showed high likelihood of unjustified transfers. Patients were more likely to be referred from non-teaching hospitals however hospital size did not seem to significantly affect unjustified transfer probability. In the pediatric group, a diagnosis of spondylosis showed high likelihood of unjustified transfers also, significant associations with odds of potentially avoidable referrals were determined for dementia cases.

As medical care in a large healthcare system concentrates in the tertiary care center so do patient referrals to the hospital. The referring physician’s discerning evaluation should consider the apparent benefit to the patient derived from an interhospital transfer. Undoubtedly, acuity of disease factors heavily into the decision, and lack of subspecialty expertise and resources at the referring institution, accepting hospital evidence-based medicine practice and patient preferences are major driving factors.

For pediatric health and stroke, the development of structured systems of care through regionalization did result in improved clinical outcomes [9, 10]. In other instances of cases diagnosed with neurological conditions, transferred patients occasionally will not derive additional treatment benefits as they might arrive in a terminal disease stage when interventions are useless, or will not undergo specialized interventions due to improved disease state or initial misdiagnosis [4]. While justified transfers often include high-risk patients or patients with associated morbidities, unjustified transfers are often occasioned at patients’ requests or for continuity of care, thus underscoring lack of subspecialty consult and remote care resources as associated factors of inappropriate patient triage at referring institutions.

As evaluation of an acute neurological condition effects neuro-critical care and advanced therapeutic approaches, temporal constraints related to disease acuity and progress denote a significant impetus for patient referral. Studies of “discharge-on-transfer” show 22.5% of transferred pediatric patients were discharged directly from the emergency department (ED), 53.3% of the cases were mostly for isolated skull fractures [11]. For non-traumatic disease, unwarranted referrals included respiratory infections, asthma and conditions without definitive referring diagnosis, *i*.*e*., fever, nausea and vomiting [12]. In our study, pediatric cranial trauma referrals included 64.6% potentially avoidable transfer cases for ages of 0–9 years and 51.0% for ages of 10–19 years however, most unwarranted transfers were determined for non-traumatic disease categories. Pediatric patients aged 10–19 years with dementia and spondylosis had the highest fraction of potentially avoidable transfers (83.3% and 100%), followed by patients aged 0–9 years diagnosed with spondylosis (80%). Both diagnoses are complex, especially pediatric dementia. The neurodegenerative and metabolic disorder presents with a myriad of symptoms, which include epilepsy (grand mal seizures), motor deterioration and visual loss (retinal degeneration). Intractable epilepsy, hypotonia, myoclonus, psychomotor development anomalies and standstill are observed in younger children. The order in which these symptoms occur varies according to age at onset. A diagnosis of dementia is extremely difficult and necessitates sophisticated laboratory and genetic tests. Such a complex disorder presenting with non-specific symptoms related to other neurological conditions can be prone to misdiagnosis. Nonetheless, pediatric dementia can be life-threatening when diagnosed and treated late, hence a timely diagnosis and referral of cases to a specialist is paramount to an improved outcome.

In young children, a suspected diagnosis of spondylosis can mask other sports-associated injuries such as spondylolysis, which frequently is a sports-associated stress fracture, thus posing a challenge for providers to diagnose a serious condition that requires advanced imaging technology and possible surgical intervention. An MRI aligned to visualize the disc might not always identify a fracture of the pars interarticularis. Smaller hospitals might not be equipped with these imaging capabilities.

In the adult cohort, both cranial and spinal trauma referrals had higher proportions of potentially avoidable transfers in the 80–89 years and over 90 years old patient groups. Published data on ED-to-ED transfers for trauma incidents show over one third of cases mostly with soft tissue, head, hand and face injuries were discharged upon arrival [13]. The authors propose specialist teleconsultation to alleviate transfer burden cost. A study on adult patients discharged to hospice after 2 days, without receiving surgical or radiological intervention, and expired patients identified 56% traumatic brain injury cases [14]. The authors conclude that judicious assessment of the benefit derived from transfer to higher level of care might improve hospital resources allocation. In our case, lack of specialized consult by a neurosurgeon and advanced diagnostic modalities (CT, MRI) at the referring institution prompted referrals of mostly non-displaced closed skull fracture cases that ultimately did not undergo surgical intervention.

For non-traumatic disease, chronic illness associated with comorbidities might factor into the referral decision. Predictive risk factors in addition to neurological deterioration are age and hydrocephalus, among others [15]. Studies on respiratory disease show lesser likelihood of transfers for patients over 65 years old [16]. Our study identified that trends in total referrals for migraine, neuro-ophthalmic disease and seizure disorders declined with advancing age but correlated with higher odds of potentially avoidable transfers across all age groups. Neurological dysfunctions with overlapping clinical patterns are often comorbid. As headache is caused by a considerable number of concurrent conditions often acute, it was perhaps not unexpected to identify migraine as a significant predictor. Seizure disorder, a frequent paroxysmal disorder similar to migraine, has many common traits. Further, basilar migraine might manifest with neuro-ophthalmic symptomatology. Perhaps the perception of different disease types associated with discrete age ranges and diseases grouped within a specific age cluster also applies herein [17]. Consideration of an acute presentation such as transient ischemic attack, occipital lobe seizures, or the possibility of underlying trauma, among other causes, masked by severe neurological episodes might impact the transfer decision prompting unwarranted referrals as neuro-radiological expertise and advanced diagnostic methodology are required for the final diagnosis. A definitive diagnosis without CT imaging is impracticable particularly for life-threatening secondary headache, which is rare compared to primary headache and usually associated with inflammation, brain mass effect and cerebrovascular disease [18]. Our data on migraine, which associated with higher unwarranted transfer probability compared to stroke and trauma, are concordant with published data on higher odds of potentially avoidable transfers for headache (OR, 3.59) compared to non-traumatic hemorrhage (OR, 0.67) [4]. However, the study identified higher odds for trauma (OR, 5.82) compared to headache. Lastly, we identified disparities in the proportion of avoidable transfers for pediatric (0–9 years) and aged (over 90 years) patients. All referrals for aged patients diagnosed with dementia, infection, psychiatric disease and primary tumor resulted in potentially avoidable transfers. Conversely, in the pediatric group all referrals for infection and psychiatric disease were justified.

Stimulation of the sensible use of teleconsultation to support judicious triage of acute status pediatric and aged patients is recommended. Published data showed that by decreasing potentially avoidable transfers in a community hospital an increase in case mix index successfully improved financial sustainability [19]. A pilot study on implementation of specialized teleconsultation screening for brain injury patients identified transfer cost reduction processes [20].

Lack of advanced directives and communication between referring and admitting physicians and deficient referring hospital infrastructure are major determinants for unwarranted transfers of complex neurological cases. In instances where patients might not derive further benefit from an interhospital transfer, palliative care at the referring institution is recommended. Also, palliative care was proposed as an optimal option for cases where advanced directives dictate forthcoming expectations, thus excluding the necessity for transfer. In stable cases where standard workup has not been completed, best medical management should address patient referral to outpatient clinic for follow-up by the specialist.

Improved infrastructure at low-volume hospitals through implementation of after-hours imaging capabilities and EEG services, and specialized consult by neuro-radiologists were also suggested. Interestingly, hospital teaching status, but not bed capacity significantly affected the probability of potentially avoidable transfers for discrete age groups.

Not-for-profit healthcare systems depend heavily on patient transfers, and to sensibly sustain their service line, healthcare systems will need to focus on the optimization of the transfer process, which will ultimately result in an increased return on investment.

## Conclusions

In this study, we have advanced our understanding of critical aspects of interhospital transfers of pediatric and adult patients diagnosed with neurological disease. Referral decisions are multifaceted. Disease acuity, complexity, and temporal constraints associated with disease progression are major determinants. In restricted patient groups, cases of neurological dysfunctions with complex, disease non-specific or overlapping symptomatology showed high likelihood of unwarranted transfers. Referring hospital infrastructure also factors into patient triage and consideration for transfer. Our data should alert clinicians treating neurological disorders in the pediatric and ageing population. Select future recommendations for judicious patient triage include improved small-sized hospital infrastructure and referral for neurological clinic follow-up.

However, our data necessitate interpretation within the limitations of the study. We have determined odds of potentially avoidable transfers within a large healthcare system with Level I Trauma Center designation. The Level I Trauma Center operates 24 hours according to established protocols, which might not reflect the dynamics of smaller healthcare systems with distinct structure and capacity for treatment. Very few hospitals have Level I Trauma Center designation. Future unwarranted transfer avoidance recommendations reflect only on referrals within our healthcare system. From data herein, we inferred that referrals from small-sized non-teaching hospitals, such as hospitals within the rural area serviced by our healthcare system, have significant odds of potentially avoidable transfers. Small-sized rural hospitals lack capabilities and resources afforded by a tertiary care center, hence recommendations should reflect only on referring units that lack select infrastructure, 24 h service, neuro specialist and tele medicine. We recognized a greater likelihood of unwarranted transfers for aged patients and complex cases with overlapping or non-specific symptomatology. As such, it is difficult to factor quality measures into the analysis. Although we did address provider recommendations related to future avoidance of unwarranted referrals, we did not assess referring unit infrastructure associated with staff-to-patient ratio. Another aspect is the population sample. Our health care system encompasses many rural area hospitals and data on the retention of patients at the referring hospital were not evaluated. We did not consider disease risk factors or case co-morbidities. Also, our study did not evaluate data on patient stabilization initiated at the referring hospital. The likelihood of unpredictable disease course progression will greatly affect the critical assessment of complicated neurological disease cases and referral decision dynamics, implicitly. Another limitation is inclusion of cases with non-specific diagnoses however, logistical reasons impressed case selection. Additionally, we grouped patient age according to an arbitrary range selection. It has been suggested that age classification should consider ranges based on specific diseases being explored and diseases should be grouped according to age-related co-occurrence, thus a more meaningful grouping of age can be derived from patterns of disease incidence [17].
